# Toddler skills predict moderate-to-late preterm born children’s cognition and behaviour at 6 years of age

**DOI:** 10.1371/journal.pone.0223690

**Published:** 2019-11-06

**Authors:** Lilly Bogičević, Marjolein Verhoeven, Anneloes L. van Baar

**Affiliations:** Child and Adolescent Studies, Utrecht University, Utrecht, Netherlands; University of Iowa, UNITED STATES

## Abstract

**Objective:**

To compare moderate-to-late preterm born (32–36 weeks’ gestation) to full term born (≥37 weeks’ gestation) children in cognitive and behavioural functioning at the age of 6 years and assess which toddler skills predict later cognitive and behavioural functioning.

**Design:**

A prospective longitudinal study with a cohort of 88 moderate-to-late preterm and 83 full term born Dutch children, followed from 18 months to 6 years of age. Orienting, alerting and executive attention skills were assessed at 18 months (corrected for prematurity), and cognitive, motor and language skills (Bayley-III-NL) at 24 months (corrected for prematurity). At 6 years (corrected for prematurity), cognitive (indices of IQ; WPPSI-III-NL) and behavioural functioning (CBCL/6-18) were assessed. Group differences and potential predictors were examined with MANCOVAs and hierarchical regression analyses.

**Results:**

At 6 years, moderate-to-late preterm born children performed poorer than full term born children on cognitive processing speed, and they showed more behavioural attention problems. Attention problems at 6 years were predicted by poorer orienting attention skills at 18 months, while lower performance IQ was predicted by poorer alerting attention skills at 18 months. Full Scale IQ and Verbal IQ at 6 years were predicted by language skills at 24 months. Moderate-to-late preterm and full term born children showed some differing correlational patterns in the associations between early skills and later functioning, although in further analyses predictors appeared the same for both groups.

**Conclusions:**

Moderate-to-late preterm born children show specific vulnerabilities at primary school-age, particularly in cognitive processing speed and behavioural attention problems. Cognitive and behavioural functioning at 6 years can be predicted by differentiated attention skills at 18 months and language skills at 24 months.

## Introduction

Preterm birth accounts for 11% of all births worldwide, of which 84% is moderate-to-late preterm (MLPT; 32–36 weeks’ gestational age (GA))[1]. MLPT children are at elevated risk of mortality[2], neonatal complications [3], and later developmental problems [4,5]compared to full term born (FT; ≥37 weeks’ GA) children. MLPT children show detectable poorer outcomes in cognitive, behavioural and educational functioning compared to FT children and most commonly reported concerns are attention problems [6–9]. Although differences in outcomes between MLPT and FT children at primary school-age are subtle and the number of MLPT children with clinical problems is relatively low, suboptimal development of these skills may still have implications for later functioning. Grade repetition and special education needs, for instance, are more common in MLPT than in FT children [9]. It is therefore important to study MLPT children’s long-term developmental course. Information on potential predictors of school-age outcomes is needed to enable earlier identification and timely deployment of prevention or intervention programs. Already during toddlerhood MLPT children show subtle delays in certain skills, such as attention and language skills [10,11], which could be precursors of later developmental problems.

Previous studies in preterm born and FT children found that attention [12,13], cognitive [14,15], language [16], and motor skills [17,18] measured at infant or toddler age were important predictors for later cognitive and behavioural outcomes, mainly in very preterm born (VPT; <32 weeks’ GA) children. Distinct predictors have not yet been identified for MLPT children in particular.

Aims of this prospective longitudinal study were 1) to compare MLPT children to FT children in cognitive and behavioural functioning at 6 years, and 2) to assess if specific attention skills, and cognitive, motor and language skills in toddlerhood predict cognitive and behavioural functioning at age 6, and if these predictors are similar for MLPT and FT children.

## Methods

### Participants

The STAP Project (Study on Attention of Preterm children) follows MLPT and FT children with a specific focus on development of attention skills. Children born between March 2010 and April 2011 were recruited from nine hospitals around Utrecht, the Netherlands. Exclusion criteria were dysmaturity (birth weight <10^th^ percentile according to Dutch reference curves) [19], multiple births, admission to a tertiary Neonatal Intensive Care Unit, severe congenital malformations, antenatal alcohol or drug abuse and chronic antenatal use of psychiatric drugs by the mother. Parents were invited to participate in the study through their paediatrician or midwife when their children were 10 months old.

**Fig 1:**
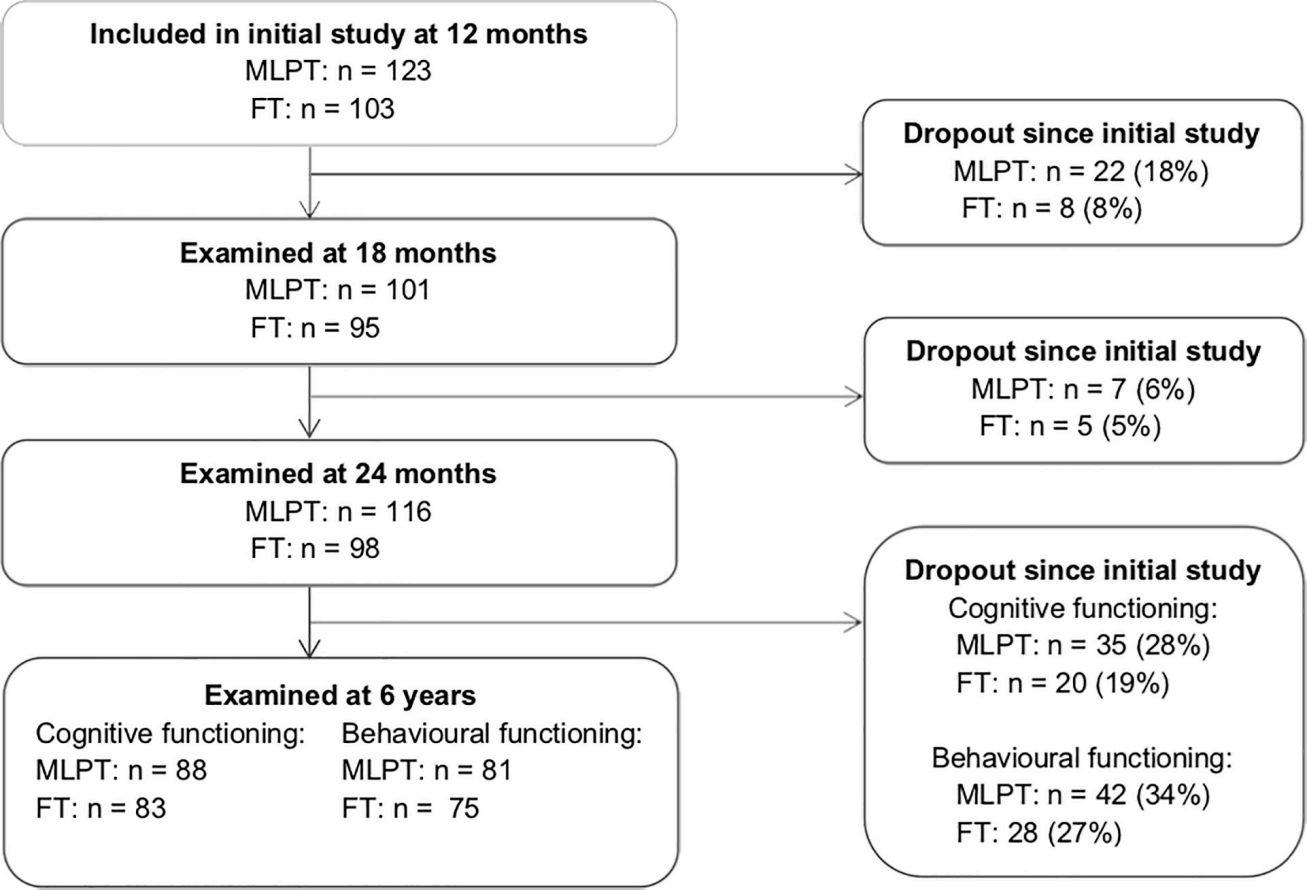
Flowchart of the inclusion procedure.

Fig 1 presents the inclusion procedure of MLPT and FT children. Overall the children who dropped out at the age of 6 years did not differ from the total sample nor their subsample (MLPT vs. FT) in terms of GA, birth weight, gender and maternal education. Neonatal and demographic characteristics of the participants are summarised in Table 1.

**Table 1:** Neonatal and demographic characteristics of the FT and MLPT groups.

	FT (n = 83)	MLPT (n = 88)
Corrected age in months at wave 1		
Mean (SD)	17.5 (0.5)	17.5 (0.5)
Range	17.0–18.0	17.0–18.0
Corrected age in months at wave 2		
Mean (SD)	23.7 (0.5)	23.6 (0.5)
Range	23.2–25.2	23.1–25.2
Corrected age in months at wave 3		
Mean (SD)	73.4 (0.6)	73.1 (0.7)*
Range	72.1–75.4	71.2–75.1
Gestational age		
Mean (SD)	39.5 (0.9)	34.7 (1.3)***
32 weeks (%)		11%
33 weeks (%)		9%
34 weeks (%)		17%
35 weeks (%)		26%
36 weeks (%)		36%
37 weeks (%)	2%	
38 weeks (%)	11%	
39 weeks (%)	30%	
40 weeks (%)	43%	
41 weeks (%)	13%	
Birth weight in grams		
Mean (SD)	3604 (455)	2529 (490)***
Range	2795–5330	1420–3850
Days in hospital		
Mean (SD)	0.4(1.1)	11.9 (10.2)***
Range	0–6	1–42
Need for oxygen^a^ (%)	0%	26%***
Phototherapy (%)	0%	35%***
Hypoglycemia (%)	0%	5%*
Gender (% boys)	45%	58%
First born (%)	49%	60%
Ethnic origin (% Dutch)	96%	96%
Maternal education (%)		
Low^b^	2%	8%
Medium^c^	10%	35%*
High^d^	88%	57%*
Maternal age at birth		
Mean (SD)	32.8(4.0)	31.3(4.4)*
Range	25–43	21–41

^a^Additional oxygen right after birth, nasal cannula and/or continuous positive airway pressure (CPAP).

^b^No education, elementary school, special education or lower general secondary education.

^c^Secondary education or vocational education.

^d^College, university or higher.

* *P* < .05

*** *P* < .001

### Procedure

MLPT children were invited at corrected age (CA) at all assessments to exclude subtle maturational effects and optimise comparison with FT children. At 18 months children were seen for assessment of attention skills. At 24 months cognitive, motor and language skills were examined. At 6 years an IQ test was administered to the child to evaluate cognitive functioning while the mother completed questionnaires. All assessments were administered by trained examiners, who were blinded for GA. The medical ethics committee of the University Medical Centre Utrecht approved this study. Written informed consent was obtained from all parents.

### Measures

Attention skills at 18 months were assessed with the Utrecht Tasks of Attention in Toddlers using Eye tracking (UTATE), an instrument specifically developed to assess distinct attention skills in toddlers by evaluating their looking behaviour [20,21]. The UTATE consists of four tasks 1) disengagement task, 2) face task, 3) alerting task, and 4) delayed response task. The disengagement task consisted of 20 trials, in which first a visual stimulus was shown at the centre of the screen, and after 2 seconds another stimulus appeared at the left or right side of the central stimulus. The face task had eight trials in which first two identical photos of child faces were presented (habituation phase), and after 8.5 seconds, one of the photos changed into a new face. This new combination was then shown for another 8 seconds. The alerting task comprised 32 trials, in which a visual stimulus was presented. In half of the trials the stimulus was preceded by a sound. In the delayed response task, the screen showed a dog hiding that went in one of two doghouses. Once the dog was hidden, a worm appeared in the centre of the screen to distract the child from the doghouses, and after a delay the child was asked to search for the dog. This task consisted of 18 trials, with the delay increasing from 0 to 10 seconds in steps of 2 seconds for every three consecutive trials [20].

From these tasks various aspects of looking behaviour were extracted to assess three latent constructs: the ability to 1) orient attention on a target, i.e. the ability to engage, disengage and shift attention focus(*orienting attention*), 2) achieve and sustain alert attention focus (*alerting attention*), and 3) plan and direct attention and inhibit behaviour (*executive attention*) [22]. The looking behaviour variables used to form the three attention constructs are described in more detail elsewhere [21]. Higher scores indicate better attention skills. Originally, these latent constructs were computed with a confirmatory factory analysis based on the 98 FT children for whom data was available at 18 months, showing good model fit [21]. Measurement invariance for this model was confirmed in the 18-month sample of 101 MLPT children [10]. Based on the 153 children for whom complete data was available at 18 months and at 6 years, we repeated this confirmatory factor analysis with the factor structure from the original model using the Lavaan package [23] in the R Project for Statistical Computing [24]. The model showed acceptable fit based on the RMSEA, CFI and TLI indices [25]: *χ*^2^ = 79.07, *P* = .002., RMSEA = .07, SRMR = .09, CFI = .96, TLI = .93.

Cognitive, motor, and language skills at 24 months were assessed with the Dutch version of the Bayley-III (Bayley-III-NL) [26]. The Bayley-III-NL consists of three indices, based on five subtests: the Cognition Index (Cognition), Motor Index (Fine Motor and Gross Motor), and Language Index (Receptive Communication and Expressive Communication). Index scores were based on Dutch norms with means of 100 and SDs of 15 with good reliability and validity [26].

Cognitive functioning at 6 years was examined with the Dutch version of the Wechsler Preschool and Primary Scale of Intelligence (WPPSI-III-NL) [27]. Four standardised indices of IQ were assessed with eight subtests of the WPPSI-III-NL. Verbal IQ (VIQ) is comprised of the subtests Information, Vocabulary and Word Reasoning. Performance IQ (PIQ) consists of Block Design, Matrix Reasoning, and Picture Concepts. Processing Speed (PSQ) consists of Symbol Search and Coding. Lastly, Full Scale IQ (FSIQ) consists of VIQ, PIQ and Coding. IQ scores were based on Dutch norms with means of 100 and SDs of 15 with good reliability and validity [27]. Norms are age-specific, with ranges of 3 months. For MLPT children two sets of IQ scores were computed based on: 1) norms for their age corrected (CA) for prematurity, and 2) norms for their chronological age, or age uncorrected (UCA) for prematurity. Clinically relevant scores were defined as IQ scores below 1 SD (≤85).

Behavioural functioning was assessed using the Dutch version of the Child Behavior Checklist (CBCL/6-18) [28], completed by mothers. The CBCL/6-18 consists of two broadband scales (internalising and externalising behaviour), and eight subscales. In the current study we used the internalising and externalising behaviour scales, and the subscale attention problems. Standardised T-scores were based on Dutch norms with good reliability and validity [28]. For the two broadband scales T-scores <60 are considered normal, 60–64 as borderline clinical, and ≥64 as clinical. For the attention problem scale, T-scores <65 are considered normal, 65–70 as borderline clinical, and ≥70 as clinical. Borderline clinical and clinical scores were considered as clinically relevant scores.

### Statistical analysis

To address our first aim, group differences in mean scores on cognitive (CA and UCA scores) and behavioural functioning at primary school-age were analysed using (Multivariate) Analyses of Covariance ((M)ANCOVAs), adjusted for maternal education. Effect sizes were assessed using partial *η*^2^ values, with .01 considered as a small effect, .06 a moderate effect, and .14 a large effect. Group differences in prevalence of clinically relevant scores for cognitive and behavioural functioning were assessed with *χ*^2^*-*tests.

To address our second aim, associations between toddler skills (predictors) and child cognitive (CA scores) and behavioural functioning at age 6 were first evaluated by Pearson’s correlations for the two groups separately. Outcome variables that significantly correlated with predictors for one or both groups were then included in multiple hierarchical regression analyses for the total sample. Considering our relatively small sample size, we analysed predictors at 18 months (orienting, alerting and executive attention skills (UTATE)) and at 24 months (cognitive, motor and language skills (Bayley-III-NL)) in separate models. In the first step we adjusted for group and maternal education. The second step contained predictors at 18 months or at 24 months. In the final step, interaction variables (group x predictor) were added to assess whether predictors differed for MLPT and FT children. To avoid multicollinearity, we centred the grand means of the predictors around 0. In each step the variance explained by the model was assessed with *R*^*2*^. The fullest model (i.e. the model with the most predictors) with a significant explained variance was used for interpretation. Cohen's *f* ^*2*^ values were used to assess effect size for each model, with a value of .02 considered as a small effect, .15 as a moderate effect, and .35 as a large effect. Considering that the second aim of the study was meant to generate rather than prove hypotheses, we did not reduce *P* values for multiple testing. Analyses were performed in IBM SPSS Statistics 25.0.

A power analysis showed that with our total sample size of 171 children who participated in this wave, we had over 80% power to detect group differences and associations of small-to-moderate effect size. For the ANCOVA used to assess group differences on FSIQ our sample size was sufficient to detect a moderate-to-large effect size.

## Results

### Functioning at 6 years

Mean scores and prevalence of clinically relevant scores for cognitive functioning are presented in Table 2. Mean group differences were adjusted for maternal education. Using UCA scores, MLPT children performed poorer on FSIQ and PSQ than FT children, with small to moderate effect sizes. Using CA scores, MLPT children still performed significantly poorer than FT children on PSQ, but not on the other cognitive measures. The effect size was moderate. Both with and without correcting for prematurity, MLPT children had a higher prevalence of clinically relevant scores for PSQ: 20% compared to 5% of FT children (*χ*^2^ = 8.67, *P* = .003).

**Table 2:** Functioning at 6 years in the FT and MLPT groups.

	Mean (SD)							% Clinically relevant scores		
	FT	MLPT (CA)	Mean Difference	Effect size	MLPT (UCA)	Mean Difference	Effect size	FT	MLPT	MLPT
	(n = 83)	(n = 85)	(95% CI)		(n = 85)	(95% CI)			(CA)	(UCA)
**Cognitive functioning**										
Full scale IQ	111.4 (12.3)	106.2 (14.2)	5.2 (1.1 to 9.2)	.01	105.1 (13.8)*	6.3 (2.3 to 10.3)	.03	0%	4%^#^	5%*
Verbal IQ^a^^,^^b^	110.2 (12.5)	105.8 (13.4)	4.4 (0.7 to 8.5)	.008	104.7 (13.5)	5.6 (1.7 to 9.6)	.06	2%	6%	9%
Performance IQ^a^^,^^b^	109.7 (12.5)	106.6 (14.3)	3.1 (-1.0 to 7.1)	.005	105.9 (14.1)	3.8 (-.0.3 to 7.8)	.02	2%	7%	8%
Processing speed IQ^a^^,^^b^	104.0 (13.3)	96.9 (15.0)**	7.1 (2.7 to 11.4)	.05	96.0 (14.8)**	7.9 (3.6 to 12.2)	.06	5%	20%**	20%**
**Behavioural functioning**										
Internalising problems^c^	45.3 (9.3)	47.8 (10.1)	-2.5 (-5.6 to 0.6)	.01				7%	10%	
Externalising problems^c^	45.7 (10.1)	46.3 (9.0)	-0.6 (-3.6 to 2.5)	.003				8%	11%	
Attention problems^c^	53.0 (4.0)	55.3 (6.3)*	-2.3 (-4.0 to -0.7)	.04				4%	12%	

^a^ MANCOVA multivariate results for MLPT (CA) vs. FT on VIQ, PIQ, PSIQ, GLC: F = 2.25, *P* = .07, partial *η*^2^ = .05.

^b^ MANCOVA multivariate results for MLPT (UCA) vs. FT on VIQ, PIQ, PSIQ, GLC: F = 3.16, *P* = .02, partial *η*^2^ = .07.

^c^ MANCOVA multivariate results for Internalising, Externalising and Attention problems: F = 2.68, *P* = .049 , partial *η*^2^ = .05.

* *P* < .05

** *P* < .01

*** *P* < .001.

Mean scores and prevalence of clinically relevant scores for mother-reported behavioural functioning are presented in Table 2. MLPT children showed significantly higher rates of attention problems than FT children after adjusting for maternal education. The effect size was small to moderate. For attention problems 12% of MLPT children showed clinically relevant scores, compared to 4% of FT children, although this difference was not statistically significant (*χ*^2^ = 3.55, *P* = .06).

### Toddler skills predicting functioning at 6 years

Correlations between toddler skills and cognitive and behavioural functioning at age 6 are shown in Table 3. Cognitive and behavioural outcomes showing significant correlations with toddler skills were further analysed in hierarchical regression analyses. Toddler’s attention skills at 18 months significantly predicted variance in PIQ and attention problems at age 6 (*R*^*2*^ = .09, *P* = .01, and *R*^*2*^ = .11, *P* = .01, respectively). Higher scores for alerting attention at 18 months predicted higher PIQ scores at 6 years for the full sample (Table 4). Lower scores for orienting attention skills at 18 months predicted higher attention problem scores at 6 years (Table 4). Effect sizes were moderate (Table 4). Models with interaction effects between group and attention skills were not significant (*R*^*2*^ = .07−.12, *P* = .08−.76); cognitive and behavioural functioning at 6 years were predicted by the same attention skills at 18 months for MLPT and FT children.

**Table 3:** Correlations between 18- and 24-months predictors and functioning at 6 years for the FT and MLPT groups.

	1.		2.		3.		4.		5.		6.		7.		8.		9.		10.		11.		12.		13	
	FT	MLPT	FT	MLPT	FT	MLPT	FT	MLPT	FT	MLPT	FT	MLPT	FT	MLPT	FT	MLPT	FT	MLPT	FT	MLPT	FT	MLPT	FT	MLPT	FT	MLPT
**18 months**																										
1. Orienting attention	-	-																								
2. Alerting attention	.84**	.84**	-	-																						
3. Executive attention	.34**	.36**	.59**	.59**	-	-																				
**24 months**																										
4. Cognitive skills	.20	.31**	.20	.30*	.11	.13	-	-																		
5. Motor skills	-.02	.08	-.07	.14	-.09	.01	.54**	.46**	-	-																
6. Language skills	.12	.09	.13	.14	-.11	.18	.40**	.45**	.39**	.29**	-	-														
**Cognitive functioning at 6 years**																										
7. Full Scale IQ	.07	.27*	.11	.33**	-.02	.27*	.33**	.11	.22*	.11	.42**	.30**	-	-												
8. Verbal IQ	.07	.19	.02	.26*	-.14	.23	.27*	.08	.12	.10	.48**	.41**	.80**	.78**	-	-										
9. Performance IQ	.12	.24*	.26*	.29*	.19	.21	.24*	.07	.20	.06	.24*	.11	.79**	.86**	.34**	.43**	-	-								
10. Processing speed IQ	-.12	.22	-.16	.25*	-.21	.18	.21	.26*	.20	.23*	.16	.23*	.43**	.57**	.21	.26*	.16	.43**	-	-						
**Behavioural functioning at 6 years**																										
11. Internalising problems	.01	-.07	.09	.06	.15	.23	-.04	-.04	-.07	.09	-.21	.13	.11	-.13	-.03	-.05	.19	-.10	.01	-.09	-	-				
12. Externalising problems	-.09	-.10	.01	.03	.13	.21	-.04	-.12	-.16	.08	-.34**	.003	-.06	-.05	-.10	-.01	-.04	-.08	.07	-.07	.47**	.47**	-	-		
13. Attention problems	-.15	-.09	-.03	.07	.08	.25*	-.07	-.09	-.04	.12	-.27*	.05	-.05	-.19	-.14	-.14	.09	-.14	-.06	-.19	.40**	.39**	.54**	.57**	-	-

* *P* < .05

** *P* < .01.

**Table 4:** Predictors of functioning at 6 years for the full sample (N = 166).

	Full scale IQ		Verbal IQ		Performance IQ		Processing speed IQ		Attention problems		Externalising problems^a^	
	*B* (95% CI)	*β*	*B* (95% CI)	*β*	*B* (95% CI)	*β*	*B* (95% CI)	*β*	*B* (95% CI)	*β*	*B* (95% CI)	*β*
**18-months predictors**												
**Step 1**												
Group^b^	-1.71 (-6.18 to 2.77)	-.06	-1.20 (-5.51 to 3.12)	-.05	-0.82 (-5.33 to 3.70)	-.03	-5.89 (-10.66 to -1.12)	-.21*	1.98 (0.23 to 3.72)	.20*		
Maternal education	5.05 (1.12 to 8.97)	.22*	5.29 (1.50 to 9.08)	.23**	3.13 (-0.84 to 7.09)	.13	1.76 (-2.41 to 5.94)	.07	0.30 (1.25 to 1.85)	.03		
*R^2^*	.06*		.06**		.02		.06*		.04			
Effect size^b^	.06		.06		.02		.06		.04			
**Step 2**												
Group^b^	-1.06 (-5.58 to 3.47)	-.04	-0.69 (-5.10 to 3.72)	-.03	-0.27 (-4.74 to 4.21)	.01	-5.46 (-10.36 to -0.57)	-.19*	1.43 (-0.34 to 3.17)	.14		
Maternal education	3.76 (-0.40 to 7.74)	.16	3.76 (-0.40 to 7.74)	.21*	1.11 (-2.94 to 5.15)	.05	1.45 (-2.95 to 5.85)	.06	0.10 (-1.49 to 1.70)	.01		
Orienting attention	-2.55 (-13.23 to 8.13)	-.08	-0.29 (-10.47 to 10.41)	-.001	-5.15 (-15.74 to 5.44)	-.15	-1.28 (-12.83 to 10.26)	-.04	-5.28 (-9.38 to -1.19)	-.42*		
Alerting attention	6.17 (-1.84 to 14.17)	.28	2.98 (-4.81 to 10.77)	.14	8.37 (0.47 to 16.27)	.39*	3.23 (-5.43 to 11.88)	.14	2.43 (-0.63 to 5.50)	.30		
Executive attention	-0.76 (-4.90 to 3.38)	-.04	-1.55 (-5.56 to 2.46)	-.08	0.40 (-3.67 to 4.46)	.02	-1.91 (-6.39 to 2.57)	-.09	1.04 (-0.58 to 2.66)	.13		
*R^2^*	.10		.08		.09*		0.07		.11*			
Effect size	.11		.09		.10		0.08		.12			
**24-months predictors**												
**Step 1**												
Group^b^	-3.11 (-7.34 to 1.13)	-.12	-2.47 (6.54 to 1.61)	-.09	-1.67 (-5.96 to 2.62)	-.06	-6.89 (-11.50 to -2.29)	-.24**	2.27 (0.49 to 4.06)	.21*	1.43 (-1.70 to 4.56)	.08
Maternal education	4.87 (1.19 to 8.56)	.21*	5.39 (1.83 to 8.95)	.24**	2.91 (-0.83 to 6.65)	.13	1.13 (-2.88 to 5.13)	.04	-0.13 (-1.68 to 1.43)	-.01	1.49 (-1.24 to 4.21)	.09
*R^2^*	.07**		.08**		.02		.06**		.05*		.01	
Effect size^b^	.08		.09		.02		.06		.05		.01	
**Step 2**												
Group^b^	-1.56 (5.62 to 2.50)	-.06	-0.68 (-4.43 to 3.06)	-.03	-0.91 (-5.23 to 3.40)	-.03	-5.92 (10.46 to -1.39)	-.20*	2.12 (0.32 to 3.92)	.20*	0.95 (-2.18 to 4.07)	.05
Maternal education	3.96 (0.40 to 7.52)	.17*	4.70 (1.41 to 7.99)	.21**	2.22 (-1.57 to 6.00)	.10	-0.20 (-4.18 to 3.77)	-.01	-0.03 (-1.62 to 1.55)	-.004	2.03 (-0.72 to 4.78)	.13
Cognitive skills	0.07 (-0.15 to 0.29)	.06	-0.01 (-0.21 to 0.19)	-.01	0.09 (-0.15 to 0.32)	.07	0.19 (-0.05 to 0.44)	.14	-0.06 (-0.15 to 0.04)	-.11	-0.11 (-0.28 to 0.07)	-.12
Motor skills	-0.02 (-0.22 to 0.18)	-.02	-0.12 (-0.29 to 0.08)	-.09	0.03 (-0.18 to 0.25)	.03	0.13 (-0.9 to 0.35)	.10	0.07 (-0.02 to 0.15)	.14	0.03 (-0.12 to 0.18)	.03
Language skills	0.35 (0.18 to 0.53)	.32***	0.49 (0.33 to 0.65)	.46***	0.14 (-0.05 to 0.33)	.13	0.11 (-.0.08 to 0.31)	.01	-0.03 (-0.11 to 0.05)	-.07	-0.10 (-0.24 to 0.03)	.07
*R^2^*	.18***		.26***		.06		.13**		.07		.05	
Effect size	.22		.37		.06		.15		.08		.05	

^a^ Because externalising problems correlated with 24-months predictors, but not with 18-months predictors, only a regression analysis with the 24-month predictors was performed.

^b^ Group: 0 = FT, 1 = MLPT.

* *P* < .05

** *P* < .01

*** *P* < .001.

Toddler skills at 24 months significantly predicted variance in FSIQ (*R*^*2*^ = .18, *P* < .001) and VIQ at age 6 (*R*^*2*^ = .26, *P* < .001). Better language skills predicted higher FSIQ and VIQ scores at 6 years for both MLPT and FT children, with a moderate-to-large and large effect size, respectively (Table 4). Models with interaction effects between group and cognitive, motor or language skills were not significant (*R*^*2*^ = .06−.30, *P* = .07−.91), when one FT child who had exceptional scores on cognitive skills at 24 months (IQ: 134) and externalising behaviour at 6 years (T-scores: 73) was excluded from the analyses.

## Discussion

This longitudinal study demonstrates that at 6 years of age, MLPT children perform poorer compared to FT children, specifically on processing speed IQ, and on mother-rated attention problems, when their scores are based on age corrected for prematurity. Therefore group differences cannot merely be explained by immaturity of the MLPT children. Our results show that even at primary school-age correcting for prematurity is important. Without correcting for prematurity, MLPT children performed significantly and substantially poorer (6 IQ points lower) on full scale intelligence than FT children. With correcting for prematurity, they still performed 2–5 IQ points lower on full scale, verbal, and performance intelligence, consistent with previous studies [6,7,9]. Studies on MLPT children older than 24 months, often do not mention whether the scores were corrected for prematurity. As even at primary school-age correcting for prematurity can show different results for corrected and uncorrected scores [29], future studies should always indicate whether age correction for prematurity was used or not.

Another important finding is that MLPT children seem to have more pronounced vulnerabilities in specific, rather than general, aspects of functioning. While previous studies assessing IQ in MLPT children did not report on their functioning on processing speed IQ [6–9], MLPT children in our study showed especially poorer processing speed (7 IQ points), consistent with findings in VPT children [30]. Moreover, MLPT children were four times more likely to show clinically relevant scores for processing speed compared to FT children (20% vs. 5%). Concerning behavioural functioning, MLPT children showed higher rates of mother-reported attention problems compared to FT children, consistent with previous studies [8,9,31]. MLPT children were also three times more likely to show clinically relevant scores for attention problems than FT children (12% vs. 4%), although this difference was not significant. Specific skills, especially various aspects of attention and processing speed, should be the focus of future research assessing neurodevelopmental difficulties in primary school-aged MLPT children, as it has already been in research concerning other subgroups of preterm born children [32–35].

Regarding the associations between early skills and long-term functioning we found different correlational patterns for MLPT and FT children. Toddler attention skills and later cognitive functioning were correlated in MLPT children, but not in FT children. Toddler cognitive and motor skills showed correlations with different outcomes for the two groups. In FT children, toddler cognitive and motor skills were correlated with later cognitive functioning (i.e. full scale, verbal and performance intelligence), with the exception of processing speed, while for MLPT children cognitive and motor skills at 24 months were correlated exclusively with later processing speed. However, these differing correlational patterns were not backed up by the regression analyses. We found no evidence that the early predictors differentially accounted for outcomes in MLPT vs. FT children. Further research with larger samples is needed to assess if distinct developmental mechanisms can be found in preterm and FT children.

Thus in our study cognitive and behavioural functioning at 6 years of age appeared to be similarly predicted in MLPT and FT children by several specific skills assessed in toddlerhood after adjusting for maternal education. Both MLPT and FT children who at 18 months had more difficulty orienting their attention, had more mother-reported attention problems at 6 years. This suggests that lower immediate visual responsiveness at toddler age is reflected in concentration difficulties at 6 years, as seen by mothers. Children who had more difficulty with alerting (i.e. sustained) attention at 18 months, showed poorer performance intelligence at primary school-age. This is in line with previous studies demonstrating that alerting attention at 7 months predicted cognitive functioning throughout early childhood in preterm born children [12], and showing that attention and processing speed skills account for lower full scale intelligence in 11-year-old preterm born children [35]. As performance intelligence tasks are often time-based and do not offer children direct feedback on their performance, children are required to sustain their focus independently. Because we aimed to generate hypotheses, it is important to replicate these findings in future studies. Nevertheless, our findings show that specific attention skills (i.e. orienting and alerting attention) at toddler age contribute to different aspects of development. Therefore, early and differentiated attention skills might be useful predictors of long-term cognitive and behavioural functioning in both MLPT and FT children.

We also found that language skills at 24 months predicted verbal intelligence and full scale intelligence (partly also representing verbal capacities) at 6 years in both MLPT and FT children, after adjusting for maternal education. This is in line with a study on VPT children showing that language skills at 18 months predicted verbal intelligence at 4.5 years [16]. The finding that language, rather than cognitive skills, predicted later cognitive functioning might indicate that language skills are more stable or more suitable for prediction from an early age, while cognitive development may follow a more complex developmental trajectory between 2 and 6 years of age. A follow-up protocol with assessment of toddlers’ language skills, as well as attention skills, might improve early identification of vulnerable children.

Finally, in our study early distinct attention skills measured at 18 months were correlated with some of the more general measures of cognitive, motor and language skills at 24 months. Future studies could evaluate which combinations of general or specific assessments, measured at the same age, show the best predictive relationships with long-term functioning. Strengths of our study include use of a recently born MLPT cohort with a control group, and a longitudinal, multi-method, multi-informant design. Our eye tracking measure provided a relatively objective and accurate measure of children’s looking behaviour and enabled us to evaluate several distinct attention skills (orienting, alerting, and executive attention) at a young age. Excluding a specific group of potentially higher-risk children by including only relatively low-risk MLPT children who did not need treatment at a NICU, allowed for a more homogeneous sample. A limitation of our sample is that most of the mothers were highly educated, especially in the FT group. Although analyses were adjusted for maternal education, generalisability to MLPT children with severe neonatal and demographic risk factors may be limited.

In conclusion, this study adds to findings of poorer functioning in MLPT born children at primary school-age and reveals vulnerabilities specifically in processing speed and attention problems, emphasising the need for assessment of specific skills. Poorer orienting attention skills at toddler age are found to be early precursors for later attention problems. As MLPT children also appear to have difficulties with processing speed, future studies should investigate early precursors for poor processing speed skills. Moreover, this study highlights the importance of distinguishing between different early developmental and attention skills, as they are uniquely related to cognitive and behavioural outcomes.
